# Nasal Reflexes

**Published:** 1890-07

**Authors:** L. M. Crichton

**Affiliations:** Clinical Assistant to Prof. Emil Gruening, New York Polyclinic and New York Eye and Ear Infirmary


VOL. VII. JULY, 1890. NO. 5.

### Original Communications.

### NASAL REFLEXES.

BY. L. M. CRICHTON, M. D.,
Clinical Assistant to Prof. Emil Gruening, New York Polyclinic and New York Eye and Ear Infirmary.

I do not invite the attention of the specialist, so much as that of the general practitioner, to what follows in this article; the subject having received much careful study at the hands of the specialist and but little from the general practitioner; far less, I think, than its importance demands.

Every physician should, in justice to his patients, become skilled enough to perform a simple rhinoscopic examination and make a diagnosis of the gross pathological conditions, even though he may not feel competent to operate or treat such cases.

Much suffering, I am sure, could be relieved and years of unhappiness prevented if the vast importance of nasal surgery were more fully appreciated by the family physician; for no class of patients are more miserable than those suffering from nasal disease and its various reflex phenomena. I have in mind now three patients, cases of extreme nasal obstruction, who threatened to end their wretched lives if they were not afforded relief.

Perhaps the most unsatisfactory and aggravating class of cases, with which a doctor comes in contact, is *headaches*. We all appreciate how exasperating it is to treat seriatim, stomach, liver, intestines, uterus, ovaries, etc.; exhaust the materia medica as well as our patience, and then get the discouraging report of "no improvement." We frequently have such patients and most heartily wish ourselves rid of them; in desperation we send him, or, as is more often the case, her, to the oculist, in hopes that "Eye strain" may be causing the trouble. Very often the asthenopia *is* the cause, and a correction of the error of refraction gives relief, but in a certain per cent. it *does not;* 'tis to this class of cases I wish particularly to draw your attention.

Through the kindness of Dr. Robert Myles, Throat and Nose Department, New York Polyclinic, and my connection with Prof. Gruening, I have been enabled to study carefully a large number of eye and nose reflexes. From among the cases I have followed I will select three as especially illustrative of *headaches* from nasal disease:

CASE I.—Miss B., æt. 21, complained of severe frontal headaches, occurring daily or every few days. Breathed with great difficulty through nose; slept poorly; voice became husky and throat felt tired towards evening; throat dry and stiff in morning on awakening.

Patient was seen by Dr. Myles January 25th. Examination showed a deflected septum, to left side, markedly thickened at point of deflection and *pressing tightly* upon middle turbinated body which was hypertrophied. Hypertrophy of posterior and of inferior turbinated body on right side. Almost absolute occlusion of left nostril.

At intervals of ten days to twelve weeks four operations were performed. One with the snare, posterior end of inferior turbinated body on right side. Two operations on the septum, one with Bosworth's nasal saw, and one with the electro-motor drill. One thorough application of chromic acid to middle turbinated body on left side.

Patient showed improvement from time of first operation. [illegible] time of writing patient reports *not having had a single headache* [illegible] *a month past ;* sleeps well ; trouble with throat disappeared a[illegible] general health markedly improved. Has taken no other tre[illegible] ment whatever since first operation and made no change in mo[illegible] of life.

CASE II.—Miss V., æt. 19, consulted Dr. Myles first part [illegible] March. Complained very much the same as case abo[illegible] Violent frontal headaches almost every day ; difficulty in na[illegible] respiration; did not sleep well; face pale and had the "worri[illegible] expression" which I have observed so frequently in this class [illegible] cases. Examination showed hypertrophy of middle and infer[illegible] turbinated bodies on left side; septum slightly deflected to l[illegible] and middle turbinated body *pressing tightly* upon it; hypertrop[illegible] of posterior end of inferior turbinated body on right side.

Three operations were performed on this case at intervals [illegible] ten days to two weeks. One thorough application of chron[illegible] acid to middle turbinated body on left side. Posterior end [illegible] inferior turbinated body on right side was snared. One appli[illegible] tion of chromic acid to inferior turbinated on left side.

Patient reported improvement decided from first operation. [illegible] time of writing patient reports having had but *one headache fo[illegible] month past*, which occurred a few days after the last operati[illegible] and was undoubtedly caused by reactive inflammation at point [illegible] operation. Nasal respiration good; sleeps well and gene[illegible] health markedly improved. Has received no other treatm[illegible] since first operation and made no change in mode of life.

CASE III.—Mr. McF., aet. about 35. Complained of m[illegible] *violent* headaches for months; slept very poorly; the almost cont[illegible] uous suffering had, as he said, " almost run him crazy, " and, [illegible] fact, his mind did seem to be somewhat affected.

Examination showed hypertrophy of right and left mid[illegible] turbinated bodies, *pressing tightly* against septum on *both* sid[illegible] Hypertrophy inferior turbinated on right side.

Two applications of chromic acid were made to reach mid[illegible] turbinated body, and one to the inferior on right side, with esult of entire cessation of the headache, and as the patient him-elf puts it, he "feels like a new man."

These operations are made painless when cocaine is *properly* ised. A four per cent. solution will not answer the purpose. The way I have seen it used and used it myself with best re-ults is as follows: a ten per cent. solution is sprayed into the nose o produce a general anæsthesia of the nasal cavity. A flattened bledget of absorbent cotton is moistened with the ten per cent. olution and then sprinkled with cocaine *crystals;* this is placed it the point to be operated, and allowed to remain about ten or ifteen minutes. The operation after this is in almost every case absolutely painless.

From the above cases and others of a similar nature I think he following deductions warranted:

That we find in the nose pathological conditions causing *narked pressure.*

That almost invariably when such conditons are found, the patient exhibits *reflex phenomena.*

That these phenomena resist all forms of general treatment.

That these pathological conditions being removed by surgical neans, the reflex phenomena cease.

That such operations are *common sense,* illustrate as clearly as anything in surgery the law of cause and effect, and are *fully warranted,* if *the diagnosis of the pathological conditions is exact, clear and correct.*

